# Novel mutations in the *USH1C* gene in Usher syndrome patients

**Published:** 2010-12-31

**Authors:** María José Aparisi, Gema García-García, Teresa Jaijo, Regina Rodrigo, Claudio Graziano, Marco Seri, Tulay Simsek, Enver Simsek, Sara Bernal, Montserrat Baiget, Herminio Pérez-Garrigues, Elena Aller, José María Millán

**Affiliations:** 1 Grupo de Investigación en Enfermedades Neurosensoriales, Instituto de Investigación Sanitaria IIS-La Fe, Valencia, Spain; 2 CIBER de Enfermedades Raras (CIBERER), Valencia, Spain; 3 U.O. Genetica Medica, Policlinico S. Orsola-Malpighi, Università di Bologna, Italy; 4 Ulucanlar Training and Research Eye Hospital, Ankara, Turkey; 5 Department of Pediatric Endocrinology, Ankara Training and Research Hospital, Ankara, Turkey; 6 Servicio de Genética, Hospital de la Santa Creu y Sant Pau. Barcelona, Spain; 7 Servicio de Otorrinolaringología, Hospital Universitario La Fe, Valencia, Spain; 8 Unidad de Genética y Diagnóstico Prenatal, Hospital Universitario La Fe, Valencia, Spain

## Abstract

**Purpose:**

Usher syndrome type I (USH1) is an autosomal recessive disorder characterized by severe-profound sensorineural hearing loss, retinitis pigmentosa, and vestibular areflexia. To date, five USH1 genes have been identified. One of these genes is Usher syndrome 1C (*USH1C*), which encodes a protein, harmonin, containing PDZ domains. The aim of the present work was the mutation screening of the *USH1C* gene in a cohort of 33 Usher syndrome patients, to identify the genetic cause of the disease and to determine the relative involvement of this gene in USH1 pathogenesis in the Spanish population.

**Methods:**

Thirty-three patients were screened for mutations in the *USH1C* gene by direct sequencing. Some had already been screened for mutations in the other known USH1 genes (myosin VIIA [*MYO7A*], cadherin-related 23 [*CDH23*], protocadherin-related 15 [*PCDH15*], and Usher syndrome 1G [*USH1G*]), but no mutation was found.

**Results:**

Two novel mutations were found in the *USH1C* gene: a non-sense mutation (p.C224X) and a frame-shift mutation (p.D124TfsX7). These mutations were found in a homozygous state in two unrelated USH1 patients.

**Conclusions:**

In the present study, we detected two novel pathogenic mutations in the *USH1C* gene. Our results suggest that mutations in *USH1C* are responsible for 1.5% of USH1 disease in patients of Spanish origin (considering the total cohort of 65 Spanish USH1 patients since 2005), indicating that *USH1C* is a rare form of USH in this population.

## Introduction

Usher syndrome (USH; OMIM 276900-2; OMIM 276905; OMIM 605472) is an autosomal recessive disorder characterized by sensorineural hearing loss, variable vestibular dysfunction, and visual impairment due to retinitis pigmentosa (RP).

Usher syndrome is the most common form of deaf-blindness of genetic origin, representing 50% of cases [1]. This disease shows a prevalence of 3.2–6.2/100,000 people [2-4].

Three clinical types of USH (types I, II, and III; USH1, USH2, and USH3) are recognized mainly on the basis of the severity and progression of hearing loss and the age of onset of RP [5]. Usher syndrome type I (USH1) is the most severe form, and it is characterized by congenital profound deafness, vestibular areflexia, and prepubertal onset of RP. To date, seven loci (USH1B-USH1H) have been mapped and five genes have been identified: myosin VIIA (*MYO7A*; USH1B), Usher syndrome 1C (*USH1C*; USH1C), cadherin-related 23 (*CDH23*; USH1D), protocadherin-related 15 (*PCDH15*; USH1F), and Usher syndrome 1G (*USH1G*; USH1G; reviewed in Saihan et al. [6]).

The causative gene, *USH1C,* was identified by positional cloning [7,8] and encodes a protein, harmonin, containing post synaptic density protein (Psd-95), Drosophila disc large tumor suppressor (DigA) and zonula occludens-1 protein (ZO1)(PDZ) domains. This gene comprises 28 exons spanning approximately 51 kb of genomic DNA. It consists of 20 constitutive exons (exons 1 to 14, 22 to 26, and 28) and eight alternatively spliced exons (15 to 21 and 27) [9].

*USH1C* mutations are the main cause of USH1 in Acadian and Quebecois patients, due to a mutation founder effect [10,11]. However, the *USH1C* gene is only involved in 7% of USH1 cases in the USA and UK populations [12] and in 6% of the French population [13].

The aim of the present work was mutation screening of the *USH1C* gene in our cohort of USH patients, to identify the genetic cause of the disease and to determine the relative involvement of this gene in USH1 pathogenesis in the Spanish population.

## Methods

### Subjects

Spanish subjects with Usher syndrome were mainly recruited from the Federación de Asociaciones de Afectados de Retinosis Pigmentaria del Estado Español (FAARPEE) and also from the ophthalmology and ear, nose, throat (ENT) services of several Spanish hospitals as part of a large study into the genetics of Usher syndrome in Spain.

This study involved 33 unrelated families that were clinically diagnosed with USH, 23 of which were diagnosed with USH1 (21 Spanish, one Italian, and one Turkish family) and ten of which were non-classified (nine Spanish and one Turkish family). Informed consent was obtained from all these patients, and this study followed the tenets of the Declaration of Helsinki.

Subjects were classified as USH1 on the basis of their clinical history and ophthalmologic, audiometric, and vestibular tests [2].

The *MYO7A*, *CDH23*, *PCDH15,* and *USH1G* genes were completely sequenced in 11 of these patients, and 19 were analyzed by microchip (Asper Biotech, Tartu, Estonia) for the molecular diagnosis of Usher syndrome. The genetic etiology of the disease in those individuals could not be determined from those previous investigations. The other three patients came to our laboratory at the time *USH1C* screening was implemented, and they had not undergone any previous screening or chip analysis.

### Mutation screening

Genomic DNA was extracted from leucocytes from peripheral blood samples. All 28 exons, including intron-exon boundaries of the *USH1C* gene, were amplified using primers previously described by Verpy et al. [8] with some modifications and standard PCR conditions (see Table 1).Amplification conditions were 95 °C 5 min followed by 35 cycles of 30 s at 95 °C, 30 s at an annealing temperature specific for each exon and 30 s at 72 °C.

**Table 1:** Primers for *USH1C* gene.

**Exon**	**Primer**	**Sequence (5′-3′)**	**Size (bp)**
1	1D-N	CGACTCAGCACCTTCGACTC	271
	1R-N	TCCGGAGTCCCAGAAGCCTG	
2	2-D	GGTGGTCTGCATAGGTCTGA	375
	2-R	TCCAGGAGCCGTGAGCATC	
3–4	3–4-D	AGTGGTCTACTCCATTCCTAA	625
	3–4-R	CCGAAGGCTCAGAAAAGTGG	
5	5-D-N	TGCCACCTGAACCTGGGATC	276
	6-R	TAGAGCCTCCAGCCAGCCTCCAC	
5–8	5–8-D-N	GAGCATCGGTGGTGAGTCTG	1191–1461 (the length of the amplicon is variable because of the presence of a VNTR in intron 5)
	5–8-R-N	TGAGGAAGGGGAGGGCAATAG	
9	9D	GGCTGAAGAGGTAGGCAGTC	376
	9R	AGGGTCAAACATCCCCAGTC	
10–12	10–12-D	CCACCAGAGCTTTCCAACTG	937
	10–12-R	ACAGCGGGCAGGAAGCAAG	
13–14	13–14-D	ATAACGTCCCCCAAAACCAA	793
	13–14-R	CACCAAGGGCTATCCATCTA	
15	15-D-N	ACCTCACAGCTCCCATGGAG	285
	15-R-N	CTGAAGCTGGGTGTCTGCAC	
16	16-D	TGTTCTGCAACCAAGGCAGG	350
	16-R	AACAGGCCAAGTCACACCATT	
17	17-D	GGCCTTCCTGTCCTAAACCTG	441
	17-R	GCTCACTCCACCCTTGTATGC	
18–19	18–19-D	CCTTGAGGGCCAGTTGGAACA	1400
	18–19-R	GAGGACATGGGAAACAGCAGT	
20	20-D	GCCGCTCAGTAGTTTCTGTG	445
	20-R	CTGCATTTTTGTCCCACCTC	
21	21-D-N	AGGGACATTGGCACGGCAG	219
	21-R-N	GAAGTGGCACAGAGTGGGAG	
22–24	22–24-D	CCATTCATCCCCCTACTCC	1092
	22–24-R	GTGGTCACCTGTTTGCTTTC	
25	25-D	TTTCAGAACCCAGGCTCAG	316
	25-R	GGCATCCTATTGTGAGACC	
26	26-D	TAGAAACGTCCTCAGACCAT	339
	26-R	GCTTGGGCCATTCCTTCAG	
27	27-D	GGAGCCCAGTGAAAGGAGAA	295
	27-R	GACGCCAGTCCAAAGAACCT	
28	28-D	TGCTCTGGCTGGGCTGAGT	628
	28-R	ATAGGGGCCACAAACCTTAT	

Exons 2, 3–4, 9, 10–12, 13–14, 16, 17, 18–19, 20, 22–24, 25, 26, 27, and 28 were amplified using primers previously described by Verpy et al. [8]. For exons 1, 5, 5–8, 15, and 21 new primers were designed.

PCR products were sequenced using manufacturer’s recommendations (Applied Biosystems, Carlsbad, CA). The sequences obtained were compared with the consensus sequence NM_153676.2 for exons 1–14 and 16–28, and with the consensus sequence NM_005709.2 for exon 15, using the BLAST program.

In those cases where mutations were detected, we performed segregation analysis. For the construction of family trees, we used Cyrillic version 2.02 software (Oxfordshire, UK).

### Computational analysis of splicing variants

To analyze the effect of variants in the splice prediction and the structure of donor and acceptor sites, in silico analyses were performed. Three programs were used: Spliceview (The National Research Council, Institute for Biomedical Technologies, Milan, Italy), NNSPLICE 0.9 from the Berkeley Drosophila Genome Project, and Human Splicing Finder, Version 2.4 (French Institute of Health and Medical Research, Inserm U827, Montpellier, France).

### Computational analysis of missense variants

The predicted effect of each missense variant was studied using three different analysis programs: Sort Intolerant From Tolerant (SIFT; J. Craig Venter Institute, San Diego, CA), which predicts whether a change is innocuous or deleterious; Pmut (Institut de Recerca Biomedica, Barcelona, Spain), which predicts if an amino acid change is neutral or pathologic; and PolyPhen (the polymorphism phenotyping program; Bork Group, Heidelberg, Germany), which estimates the consequence of an amino acid substitution as being possibly deleterious or deleterious.

## Results

### Mutation analysis

Mutation screening was performed on members of 33 USH families. As a result, two clearly pathogenic, novel mutations were identified in the *USH1C* gene.

**Figure 1:**
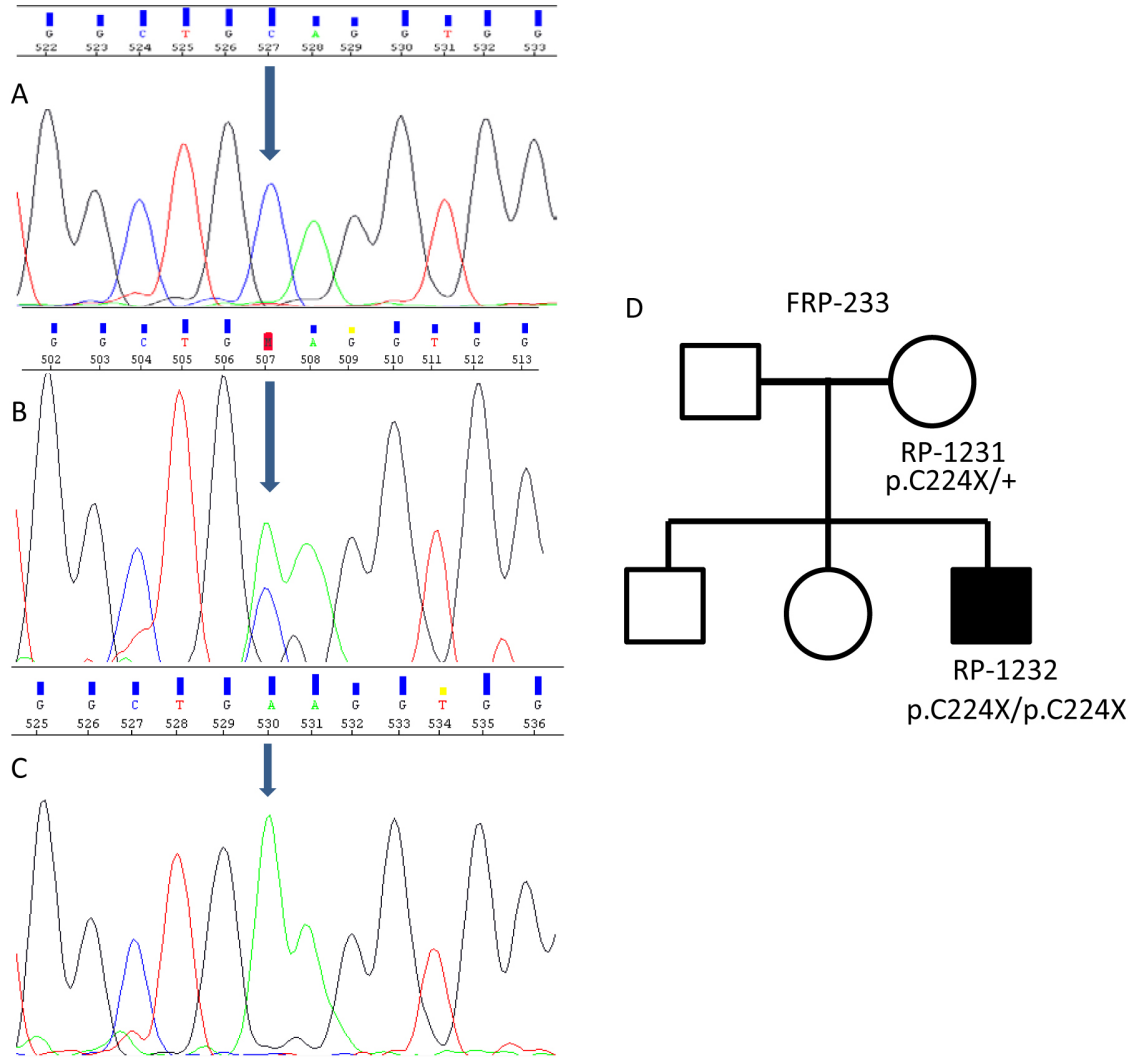
Segregation analysis of the mutation c.672C>A (p.C224X) identified in family FRP-233. **A**: Electropherogram corresponding to the wild type sequence (c.672C). **B**: Electropherogram corresponding to the healthy mother, carrying the mutation in heterozygous state (c.672C>A). **C**: Electropherogram corresponding to the patient, carrying the mutation in homozygous state (c.672A). Blue arrow indicates position c.672 is in **A**, **B** and **C**, **D**: Family tree showing the segregation of the p.C224X mutation.

The first novel mutation identified was a non-sense mutation in exon 8, p.C224X (c.672C>A). This mutation was found in a homozygous state in a Spanish USH1 patient (family FRP-233; patient RP-1232). Segregation analysis showed that the patient’s healthy mother carried the mutation in a heterozygous state (Figure 1).

**Figure 2:**
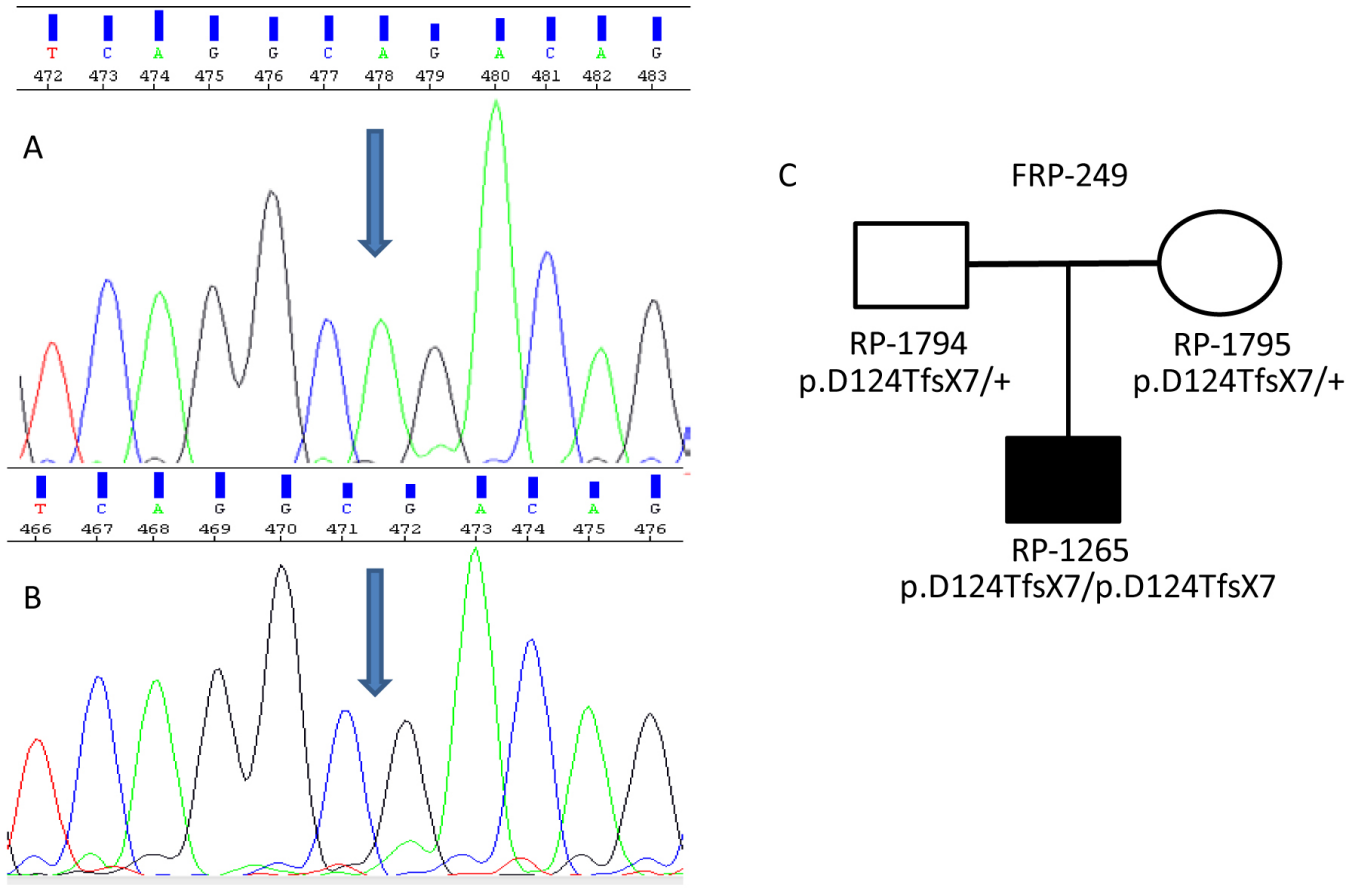
Segregation analysis of the mutation c.369delA (p.D124TfsX7) identified in family FRP-249. **A**: Electropherogram corresponding to the wild type sequence (blue arrow indicating c.369A nucleotide position). **B**: Electropherogram corresponding to the patient, carrying the mutation in homozygous state (c.369delA). **C**: Family tree showing the segregation of the p.D124TfsX7 mutation.

The second novel mutation was a frame-shift mutation, p.D124TfsX7 (c.369delA). This mutation was identified in an Italian USH1 patient (family FRP-249; patient RP-1265) in exon 4 in a homozygous state. Segregation analysis confirmed that this mutation co-segregates with the disease, and this deletion was detected in the patient’s healthy parents in a heterozygous state (Figure 2).

*USH1C* gene mutation screening allowed us to detect 47 additional sequence variants, 25 of which had previously been reported as presumably non-pathogenic (see Table 2).

**Table 2:** Novel DNA sequence variants in the *USH1C* gene identified from our cohort of 33USH patients.

**Exonic variants**				
**Exon**	**Nucleotide change**	**Amino acid change**	**SNPs**	**Allele frequency**
7	c.569C>T	p.S190L		1/66
14	c.1136G>A	p.G379D		1/66
**Intronic variants**				
**Intron**	**Nucleotide change**		**SNPs**	**Allele frequency**
2	c.104+23T>C			5/66
	c.105–54T>G			4/66
5	c.496+33A>G		rs12795083	8/66
	c.496+66G>T		rs45552041	5/66
	c.497–104A>G			6/66
	c.497–72G>T		rs28671305	4/66
7	c.580–51T>C		rs36001077	4/66
9	c.760–66T>C		rs4757539	19/66
10	c.819+10G>C		rs41282936	1/66
	c.819+66A>G			3/66
13	c.1086–12G>A		rs11024318	2/66
25	c.2490+56G>C			1/66
	c.2491–100C>G			1/66
26	c.2547–21T>C			1/66
	c.2547–11T>C		rs10832795	26/66
27	c.2656–47C>T		rs2072225	9/66
28	c.3141+215A>G			25/66
	c.3141+190C>T			25/66
	c.3141+49T>C			24/66
	c.*420_423delAACA 3′UTR			2/66

Two novel missense variants, p.S190L and p.G379D, were identified in our cohort with a low allele frequency. We performed computational analysis with the programs SIFT, PolyPhen, and Pmut to infer the pathologic effect of these variants. These programs generated contradictory results. The SIFT program predicted that the substitution of a serine for a lysine in codon 190 (p.S190L) of the protein would affect its function (score of 0.02; normalized probabilities less than 0.05 are predicted to be deleterious; those greater than or equal to 0.05 are predicted to be tolerated), but that the change from glycine to aspartic acid in codon 379 (p.G379D) would not alter protein function (score of 0.43). Conversely, the program Pmut predicted that change p.S190L would not alter protein function, but that change p.G379D was pathologic. Finally, the program PolyPhen predicted that both substitutions possibly affected protein function.

**Figure 3:**
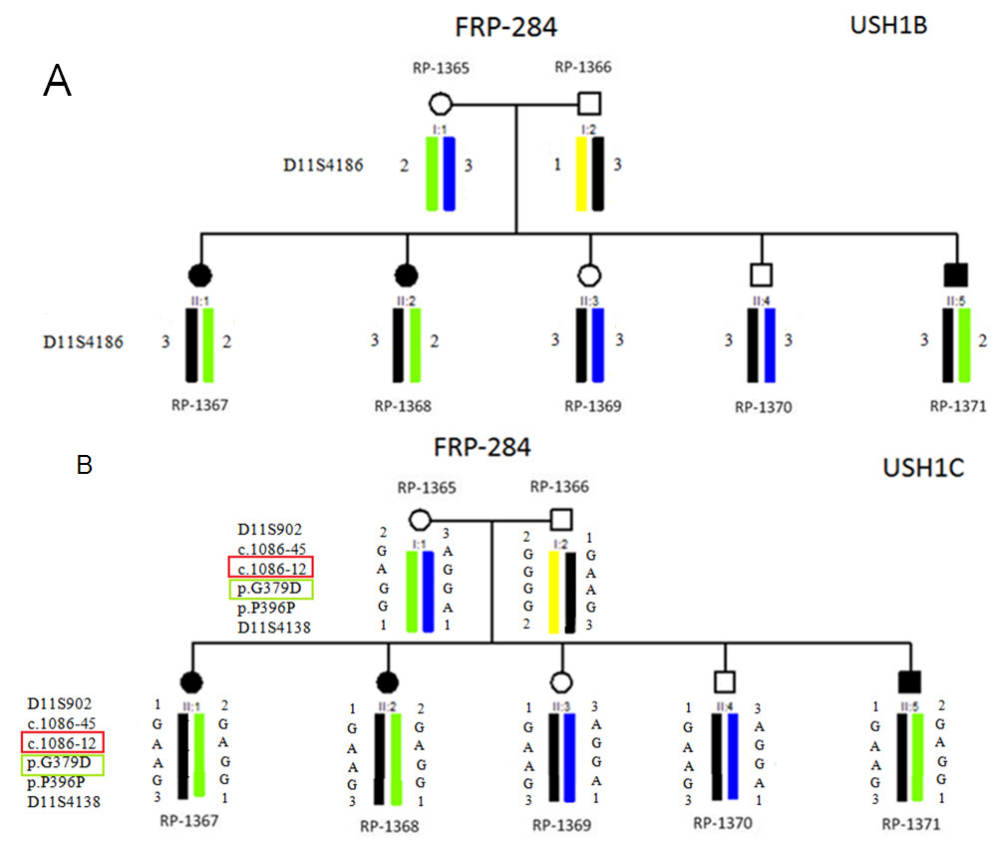
Linkage analysis for the USH1B and USH1C loci in the family FRP-284. **A**: Linkage analysis for the USH1B locus. The three affected patients of this family (RP-1367, RP-1368 and RP-1371) shares the same haplotype. For this locus, only one informative marker has been used (D11S4186). The distance of this marker to the *MYO7A* gene is 42,161 bp. **B**: Linkage analysis for the USH1C locus. The three affected patients of this family (RP-1367, RP-1368 and RP-1371) shares the same haplotype. For this locus we used two informative extragenic markers (D11S902 and D11S4138) and two intragenic markers (c.1086–45G>A and p.P396P). The distances from D11S902 and D11S4138 to the *USH1C* gene are 26,790 bp and 189,788 bp, respectively. The segregation analysis for the variants c.1086–12G>A (red) and p.G379D (green) is also shown.

An intronic variant, c.1086–12G>A, was identified in a homozygous state in three affected patients of one Turkish family (FRP-284; patients RP-1367, RP-1368, and RP-1371). This family was described as clinically compatible with USH2 by Simsek et al. [14]. However, we performed a linkage analysis for all known loci so far implicated in the Usher syndrome (data not shown), and we were able to discard linkage to all of them except for USH1B and USH1C (Figure 3). The haplotype analysis of the USH1B locus was limited because it was based only on one informative marker. However, the involvement of this locus was reduced by mutation screening of the *MYO7A* gene [15]. Thus, we included this family in the *USH1C* mutation screening. The analyses of predictions of the effects of the variant c.1086–12G>A indicated that the acceptor site would not be recognized (Spliceview) and that the score for acceptor site recognition would be reduced slightly from 48 to 45 and from 83.79 to 83.6 for NNSPLICE and Human Splicing Finder, respectively. In addition, we looked for this change at the NCBI dbSNP. This variant (rs11024318) has an allele frequency of G (98.3%) and A (1.7%) in the European population (HapMap-Ceu/ss52068547), and it is present in a homozygous state in 8.7% of an African-American population (AFD_EUR_PANEL/ss23639768) and in 6.1% of a sub-Saharan African population (HapMap-YRI/ss52068547).

In the present work, we performed a mutation screening of the *USH1C* gene by direct sequencing in 33 USH patients. Thirty of the individuals were of Spanish origin; 21 of them were clinically USH1, but we could not obtain sufficient clinical data to classify the remaining nine individuals. As a result, we only detected two clearly pathogenic mutations in two USH1 families of Italian and Spanish origins. These results indicate that mutations in *USH1C* are responsible for 1.5% of incidences of USH1 in patients of Spanish origin (considering the total cohort of 65 Spanish USH1 patients that have been studied since 2005 for the five USH1 genes [16-19]). This would suggest that *USH1C* is a rare form of USH in this population.

## Discussion

To date, few mutation screenings for the *USH1C* gene have been performed. Among them, only the studies performed by Ouyang et al. [12] and Roux et al. [13] were formal molecular epidemiological studies. These authors reported the *USH1C* gene as responsible for the disease in 7% of cases in a cohort of UK and USA patients, and in 6% of the French USH1 population, respectively.

Eleven clearly pathogenic mutations have so far been described in the USH1C gene [20]. These mutations are distributed along the entire gene, without the existence of hot spots.

In the present work, the two novel mutations, p.C224X and p.D124TfsX7, were identified in a homozygous state—the former in a Spanish family and the latter in an Italian family. Both families were diagnosed as USH1, and both possessed a possible consanguineous background, since the parents of the patients came from small villages in Spain and Sardinia.

Two novel missense variants, p.S190L and p.G379D, were found in a heterozygous state. The p.S190L variant was found in the same patient (RP-1232) that possessed mutation p.C224X in a homozygous state, although in this patient, mutation p.C224X alone was sufficient to cause the disease. The p.G379D variant was identified in three affected members of the same Turkish family (RP-1367, RP-1368, and RP-1371), where we also found the nucleotide change c.1086–12G>A (Figure 3B). The nucleotide change c.1086–12G>A is predicted to abolish a splicing site, based on results from the in silico analysis program, Spliceview. However, we found this change as an SNP (rs11024318) in the NCBI SNP database. Thus, the predicted splicing effect is not evidence for a pathologic effect of this variant.

The results of this study show that the *USH1C* gene has a very low mutation prevalence compared with other USH1 genes. In the total cohort of 65 Spanish USH1 patients, the *MYO7A* gene is involved in 35.4% of cases, *CDH23* in 15.4%, *PCDH15* in 10.8%, and *USH3A* in 3%. No mutations have been described from *USH1G* ( [15-19] and data not shown). The *USH1C* gene mutations represent 1.5% of cases.

Thus, 33.8% of our USH1 patients remain genetically uncharacterized. We cannot exclude (1) the possibility of the presence of large rearrangements undetectable by PCR, as has been reported for the *PCDH15* gene [21,22] (2) the presence of mutations responsible for clinical type I in USH2 genes (3) the presence of mutations located in promoter regions or introns far from the consensus sequences of splicing and (4) mutations associated with USH1 in still unknown genes in these unsolved cases. The genes coding for proteins involved in the “Usher protein network” and other genes with expression profiles in the retina and inner ear are excellent candidates. Consequently, mutation screening of these genes will facilitate determination as to whether some are implicated in the disease.

## References

[1] Vernon M (1969). Sociological and psychological factors associated with hearing loss.. J Speech Hear Res.

[2] Hope CI, Bundey S, Proops D, Fielder AR (1997). Usher syndrome in the city of Birmingham-prevalence and clinical classification.. Br J Ophthalmol.

[3] Kimberling WJ, Weston MD, Möller C, van Aarem A, Cremers CW, Sumegi J, Ing PS, Connolly C, Martini A, Milani M, Tamayo ML, Bernal J, Greenberg J, Ayuso C (1995). Gene mapping of Usher syndrome type IIa: localization of the gene to a 2.1 –cM segment on chromosome 1q41.. Am J Hum Genet.

[4] Espinós C, Millan JM, Beneyto M, Najera C (1998). Epidemiology of Usher syndrome in Valencia and Spain.. Community Genet.

[5] Davenport SLH, Omenn GS. The heterogeneity of Usher Syndrome. Amsterdam Excerpta Media Foundation. International Congress ser abstr. 1977; 215:87–88.

[6] Saihan Z, Webster AR, Luxon L, Bitner-Glindzicz M (2009). Update on Usher syndrome.. Curr Opin Neurol.

[7] Bitner-Glindzicz M, Lindley KJ, Rutland P, Blaydon D, Smith VV, Milla PJ, Hussain K, Furth-Lavi J, Cosgrove KE, Shepherd RM, Barnes PD, O'Brien RE, Farndon PA, Sowden J, Liu XZ, Scanlan MJ, Malcolm S, Dunne MJ, Aynsley-Green A, Glaser B (2000). A recessive contiguous gene deletion causing infantile hyperinsulinism, enteropathy and deafness identifies the Usher type 1C gene.. Nat Genet.

[8] Verpy E, Leibovici M, Zwaenepoel I, Liu XZ, Gal A, Salem N, Mansour A, Blanchard S, Kobayashi I, Keats BJ, Slim R, Petit C (2000). A defect in harmonin, a PDZ domain-containing protein expressed in the inner ear sensory hair cells, underlies Usher syndrome type 1C.. Nat Genet.

[9] Zwaenepoel I, Verpy E, Blanchard S, Meins M, Apfelstedt-Sylla E, Gal A, Petit C (2001). Identification of three novel mutations in the USH1C gene and detection of thirty-one polymorphisms used for haplotype analysis.. Hum Mutat.

[10] Savas S, Frischhertz B, Pelias MZ, Batzer MA, Deininger PL, Keats BB (2002). The USH1C 216G→A mutation and the 9-repeat VNTR(t,t) allele are in complete linkage disequilibrium in the Acadian population.. Hum Genet.

[11] Ebermann I, Lopez I, Bitner-Glindzicz M, Brown C, Koenekoop RK, Bolz HJ (2007). Deafblindness in French Canadians from Quebec: a predominant founder mutation in the USH1C gene provides the first genetic link with the Acadian population.. Genome Biol.

[12] Ouyang XM, Yan D, Du LL, Hejtmancik JF, Jacobson SG, Nance WE, Li AR, Angeli S, Kaiser M, Newton V, Brown SD, Balkany T, Liu XZ (2005). Characterization of Usher syndrome type I gene mutations in an Usher syndrome patient population.. Hum Genet.

[13] Roux AF, Faugère V, Le Guédard S, Pallares-Ruiz N, Vielle A, Chambert S, Marlin S, Hamel C, Gilbert B, Malcolm S, Claustres M, French Usher Syndrome Collaboration. (2006). Survey of the frequency of USH1 gene mutations in a cohort of Usher patients shows the importance of cadherin 23 and protocadherin 15 genes and establishes a detection rate of above 90%.. J Med Genet.

[14] Simsek T, Ozdamar Y, Simsek E, Men G (2009). Usher Syndrome Associated With a Variant of Dandy-Walker Malformation.. J Pediatr Ophthalmol Strabismus.

[15] Jaijo T, Aller E, Beneyto M, Najera C, Graziano C, Turchetti D, Seri M, Ayuso C, Baiget M, Moreno F, Morera C, Perez-Garrigues H, Millan JM (2007). MYO7A mutation screening in Usher syndrome type I patients from diverse origins.. J Med Genet.

[16] Jaijo T, Aller E, Oltra S, Beneyto M, Nájera C, Ayuso C, Baiget M, Carballo M, Antiñolo G, Valverde D, Moreno F, Vilela C, Perez-Garrigues H, Navea A, Millán JM (2006). Mutation profile of the MYO7A gene in Spanish patients with Usher syndrome type I.. Hum Mutat.

[17] Jaijo T, Aller E, García-García G, Aparisi MJ, Bernal S, Avila-Fernández A, Barragán I, Baiget M, Ayuso C, Antiñolo G, Díaz-Llopis M, Külm M, Beneyto M, Nájera C, Millán JM (2010). Microarray-based mutation analysis of 183 Spanish families with Usher syndrome.. Invest Ophthalmol Vis Sci.

[18] Oshima A, Jaijo T, Aller E, Millan JM, Carney C, Usami S, Moller C, Kimberling WJ (2008). Mutation profile of the CDH23 gene in 56 probands with Usher syndrome type I.. Hum Mutat.

[19] Aller E, Jaijo T, Beneyto M, Nájera C, Morera C, Pérez-Garrigues H, Ayuso C, Millán J (2007). Screening of the USH1G gene among Spanish patients with Usher syndrome. Lack of mutations and evidence of a minor role in the pathogenesis of the syndrome.. Ophthalmic Genet.

[20] Baux D, Faugère V, Larrieu L, Le Guédard-Méreuze S, Hamroun D, Béroud C, Malcolm S, Claustres M, Roux AF (2008). UMD-USHbases: a comprehensive set of databases to record and analyse pathogenic mutations and unclassified variants in seven Usher syndrome causing genes.. Hum Mutat.

[21] Le Guédard S, Faugère V, Malcolm S, Claustres M, Roux AF (2007). Large genomic rearrangements within the PCDH15 gene are a significant cause of USH1F syndrome.. Mol Vis.

[22] Aller E, Jaijo T, García-García G, Aparisi MJ, Blesa D, Díaz-Llopis M, Ayuso C, Millán JM (2010). Identification of large rearrangements of the PCDH15 gene by combined MLPA and a CGH: large duplications are responsible for Usher syndrome.. Invest Ophthalmol Vis Sci.

